# Fatal Neonatal Echovirus 11 Infection Following Maternal Peripartum Illness: A Case Report with Literature Review

**DOI:** 10.1055/a-2764-2405

**Published:** 2025-12-30

**Authors:** Ming-Ju Wang, Chia-Chen Lee, Hung-Yang Chang, Chie-Pein Chen

**Affiliations:** 1 Department of Obstetrics and Gynecology, MacKay Memorial Hospital, Taipei, Taiwan; 2 Department of Pediatrics, MacKay Children's Hospital, Taipei, Taiwan; 3 Department of Medicine, MacKay Medical University, New Taipei City, Taiwan

**Keywords:** Echovirus 11, neonatal sepsis, vertical transmission, fulminant hepatitis

## Abstract

Neonatal Echovirus 11 (E-11) infection acquired perinatally often manifests as a severe, sepsis-like illness with rapid clinical deterioration within the first week of life. The infection is characterized by fulminant multiorgan failure, commonly involving severe hepatitis with coagulopathy, myocarditis, and meningoencephalitis. Adverse outcomes are strongly associated with prematurity and vertical transmission from a mother with an acute peripartum infection, which precludes the transfer of protective serotype-specific antibodies. We present a fatal case of E-11 sepsis in a late preterm neonate following maternal peripartum illness.

## Introduction

Neonatal sepsis remains a significant cause of morbidity and mortality, with viral pathogens like Enteroviruses playing a crucial role. 1 2 Echovirus 11 (E-11), a nonpolio enterovirus, is particularly notorious for causing severe, often fatal, illness in newborns, characterized by rapid onset of multiorgan failure including fulminant hepatitis, myocarditis, coagulopathy, meningoencephalitis, and sepsis syndrome. 1 2 3 4 5 6 Transmission can occur in-utero, intrapartum, or postnatally. 1 2 7 8 We present a late preterm neonate who developed fulminant E-11 sepsis with extensive multiorgan involvement, following maternal peripartum fever and other symptoms consistent with viral illness, and in the context of known enterovirus exposure within the family reported shortly after delivery.

## Case

A 31-year-old female, Gravida 2, Para 1, presented to the emergency department at 36 ^5/7^ weeks of gestation with a fever reaching 38.8°C and whole abdominal pain. Her prenatal care had been unremarkable. Initial evaluation revealed upper abdominal tenderness. Laboratory investigations showed a leukocytosis of 17,200/μL with a left shift and a mildly elevated C-reactive protein (CRP) level of 1.68 mg/dL. An obstetric consultation confirmed active labor with a breech presentation, necessitating an emergency cesarean section.

A female infant was delivered with a birth weight of 2,555 g and Apgar scores of 9 at 1 and 5 minutes. The mother's postoperative course included transient chest tightness, one episode of watery diarrhea, and a single febrile episode with a slight cough. The day after delivery, she informed the medical team that her older daughter had recently been clinically diagnosed with an enterovirus infection at a local clinic.

The infant was admitted to the Newborn Baby Centre for transient tachypnea of the newborn. A sepsis work-up was initiated, and initial laboratory data including a complete blood count and CRP, were within normal limits. Empiric antibiotics (ampicillin and gentamicin) were administered. Her respiratory distress gradually improved, and she was weaned to room air by the third day of life.

However, on day 3, the infant acutely deteriorated with a fever of 38.3°C, accompanied by episodes of apnea and bradycardia. She exhibited increased work of breathing, dyspnea with cyanosis, and bilateral rales on auscultation. Physical examination revealed developing ecchymosis over both legs and hepatosplenomegaly. A chest radiograph showed bilateral lung field haziness. Given the rapid deterioration, maternal peripartum illness, and household exposure, neonatal enteroviral infection was strongly suspected. The viral RNA was extracted from the infant's throat swab, cerebrospinal fluid, and rectal swab specimens using the QIAmp Viral RNA mini Kit (QIAGEN, Germantown, MD). The samples were tested using a reverse transcription-seminested PCR (RT-snPCR) assay targeting the VP1 gene for the detection and identification of enterovirus RNA as previously described. 9 The sequence of the enterovirus VP1 capsid gene correlates with the serotype. The enterovirus PCR was positive, and subsequent nucleotide sequencing confirmed the pathogen as E-11. 9

The infant's condition progressed to fulminant sepsis with multiorgan failure, presenting as a classic neonatal hemorrhage–hepatitis syndrome, characterized by severe coagulopathy, developing ecchymosis, and fulminant hepatitis. She required intubation and mechanical ventilation for respiratory failure. Severe hypotension ensued, requiring multiple vasoactive infusions (dopamine, dobutamine, norepinephrine, and vasopressin). Severe coagulopathy consistent with disseminated intravascular coagulation (DIC) was managed with vitamin K1, fresh frozen plasma, and platelet transfusions. She also developed seizures and anuric acute kidney injury, requiring continuous veno-venous hemofiltration (CVVH).

**Fig. 1:**
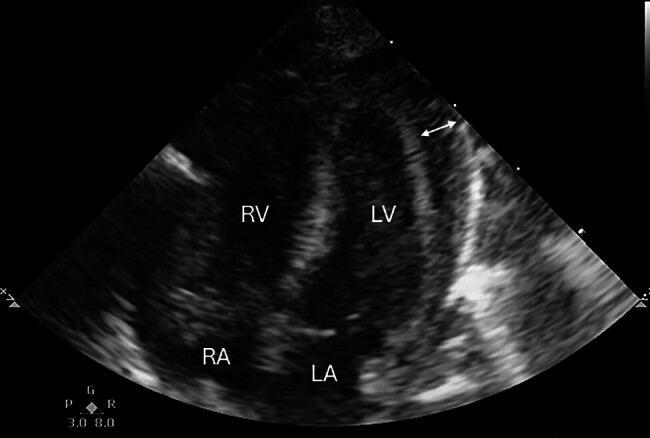
Apical 4-chamber view of echocardiogram showing the presence of pericardial effusion (pericardial effusion indicated by arrow). LA, left atrium; LV, left ventricle; RA, right atrium; RV, right ventricle.

Intravenous immunoglobulin (IVIG) at a dose of 1g/kg and methylprednisolone pulse therapy were administered. Despite maximal intensive care, her condition remained critical, characterized by refractory shock and progressive multiorgan failure. This refractory shock was attributed to acute myocarditis. The diagnosis was based on echocardiographic findings—global myocardial dysfunction, decreased left ventricular ejection fraction of 45.5%, and pericardial effusion ( Fig. 1 )—combined with a rapid rise in serum troponin-I from 0.016 to 9.228 ng/mL. The extracorporeal membrane oxygenation (ECMO) team was consulted but determined she was not a suitable candidate due to the severity of the established multiorgan damage. After comprehensive discussions with the family regarding the poor prognosis, a decision was made for withdrawal of life support. The infant passed away on the sixth day of life. The cause of death was attributed to E-11 sepsis complicated by acute fulminant hepatitis, severe coagulopathy/DIC, myocarditis with refractory shock, acute kidney injury, and respiratory failure.

## Discussion

E-11, a serotype belonging to the Enterovirus B species, is a globally recognized pathogen capable of causing severe, life-threatening infections in the neonatal population. 10 While often causing mild disease, E-11 is frequently implicated in outbreaks and sporadic cases of fulminant sepsis-like illness. 10 11 12 The case presented here, involving rapid deterioration to multiorgan failure and death in a late preterm infant, aligns with the recognized severe potential of this serotype. To provide a broader context, Table 1 synthesizes outcomes from selected literature.

**Table 1:** Reported outcomes of confirmed echovirus 11 infections in neonates from literature

Study (y)	Case/cohort	Transmission and onset	Key clinical features	Outcome (mortality)
Index case (2025)	Late preterm female (GA 36 ^5/7^ wk)	Intrapartum/perinatal (maternal fever) Onset: day 3	Fulminant sepsis, hepatitis, myocarditis, coagulopathy, AKI	Fatal
Grapin et al. (2023) 3	9 preterm infants (GA 31 ^6/7^ ∼36 ^3/7^ wk)	Intrapartum/perinatal (maternal illness) Onset: 3–5 d	Severe sepsis, fulminant hepatitis/coagulopathy, AKI	7/9 (78%) fatal
Perniciaro et al. (2025) 13	Late preterm male twins (GA 35 ^5/7^ wk)	Intrapartum/perinatal (maternal swab + ) Onset: day 7	Twin A: septic shock, severe hepatitis; Twin B: asymptomatic	1/2 (50%) fatal
Modlin (1980) 6	4 preterm infants	Intrapartum/perinatal (maternal illness) Onset: 4–6 d	Jaundice, progressive hepatic failure, bleeding	4/4 (100%) fatal
Modlin (1986) 1	Review of 43 cases	Intrapartum/perinatal (maternal illness <1wk prior) Onset: 3–5 d	Severe hepatitis, CNS infection	83% mortality with hepatitis
Hirade et al. (2023) 4	Term infant	In-utero (maternal fever 12 d prior; placental RNA + ) Onset: at birth	Fulminant liver failure, DIC, HLH, adrenal necrosis	Fatal
Willems et al. (2006) 14	Late preterm infant (GA 36 ^5/7^ wk)	In-utero (maternal fever at delivery) Onset: 30-min postbirth	Pneumonia, PPHN	Fatal
Tassin et al. (2014) 7	Term infant	In-utero (maternal illness at 12-wk GA) Onset: at birth	Extreme pulmonary hypoplasia	Fatal
Ho et al. (2020) 8	10 neonates	Postnatal/nosocomial, onset: mean 21.5 d	Mostly fever; 1 case with severe hepatitis	All survived

Abbreviations: AKI, acute kidney injury; DIC, disseminated intravascular coagulation; GA, gestational age; HLH, hemophagocytic lymphohistiocytosis; PPHN, persistent pulmonary hypertension of the newborn.

The collated data in Table 1 underscore a broad spectrum of clinical outcomes, ranging from asymptomatic infection to rapidly progressive multiorgan failure with high mortality. 8 13 The clinical presentation of severe neonatal E-11 typically manifests within the first week of life, often between days 3 and 7, sometimes following a biphasic course. 1 3 6 The initial presentation frequently mimics bacterial sepsis, including temperature instability, lethargy, poor feeding, and respiratory distress. Rapid progression to multiorgan dysfunction is characteristic of severe disease, with fulminant hepatitis, coagulopathy, and myocarditis being particularly severe hallmarks. 2 Based on the patterns observed in these cases and the broader literature, the mode of transmission is a key determinant of clinical presentation and outcome, as summarized below.

Table 2 synthesizes the key distinctions between the different transmission routes, highlighting how the timing of infection and the presence of maternal antibodies critically shape the clinical outcome. Intrapartum or late perinatal transmission, which occurs when the mother is infected within 1 to 2 weeks of delivery, is the more frequently reported route for severe neonatal disease. 1 15 In this scenario, the infant is born without sufficient maternal antibodies and, after an incubation period of 3 to 7 days, develops the classic fulminant sepsis-like illness described previously, which carries the highest reported mortality rates. 1 2 3 In contrast, true congenital (in-utero) infection is rarer and results from transplacental infection. This can lead to catastrophic outcomes before birth, such as fetal demise or stillbirth. 7 If the pregnancy continues, the neonate may be born already critically ill with multiorgan failure, presenting with symptoms on the first day of life—a distinct clinical timeline from the more common perinatal transmission route. 4 Late congenital infection can also affect the lungs; one reported case involved a newborn with severe pneumonia and persistent pulmonary hypertension, resulting in death within 36 hours. 14 The two forms of vertical transmission, while both severe, present differently. Finally, postnatal transmission occurs horizontally after birth, often in a nosocomial setting or from family members. 8 This route generally leads to a much milder clinical course with a later onset of symptoms, typically after the first week of life. 8 The lower severity is attributed to the fact that these infants have usually received a protective baseline of passive maternal IgG antibodies in utero, prior to their postnatal exposure to the virus. 1 8 Consequently, mortality is significantly lower compared to vertical transmission routes.

**Table 2:** Comparison of characteristics between neonatal echovirus 11 transmission routes

Characteristics	In-utero (congenital)	Intrapartum/perinatal	Postnatal
Timing of maternal infection	Weeks to months predelivery	Within 1–2 wk before delivery	Postdelivery contact
Maternal antibodies (IgG)	Fetal damage before IgG transfer	Insufficient time for protective IgG transfer	Neonate usually has passive immunity
Neonatal symptom onset	At birth or within days	Typically 3–7 d	Variable, often > 7 days
Typical severity	Fetal loss, stillbirth, severe anomalies	Most severe: sepsis, fulminant hepatitis/myocarditis	Often asymptomatic or mild
Prognosis/mortality	Poor if symptomatic at birth	Highest reported mortality	Generally lower than vertical transmission

The case presented in this report serves as a classic illustration of the principles of intrapartum/perinatal transmission. The maternal fever and abdominal pain at the time of delivery placed the infection squarely within the high-risk peripartum window, preventing the transfer of protective antibodies. The infant's clinical course followed the archetypal trajectory for this transmission route: an initial period of relative stability for 2 days, followed by a sudden, catastrophic deterioration on the third day of life, consistent with the typical 3- to 7-day incubation period. The subsequent rapid progression to fulminant hepatitis, severe coagulopathy, and multiorgan failure perfectly mirrors the “hemorrhage–hepatitis syndrome” that defines severe, perinatally acquired E-11 disease ( Supplementary Table S1 for diagnostic criteria [available in online version only]). This case properly connects the literature to clinical reality, demonstrating how the specific timing of maternal illness directly translates to a fatal outcome in the immunologically vulnerable neonate.

While the transmission route provides the fundamental framework, several additional factors can modulate the clinical course. Prematurity is a consistently identified risk factor for severe outcomes, due to both immune immaturity and lower levels of passively acquired IgG. 3 6 A strong male predominance has been observed in recent severe outbreaks, suggesting a possible X-chromosome-linked genetic susceptibility. 3 13 16 The most profound insight comes from discordant twins, where one twin suffers a fatal infection, whereas the other remains asymptomatic despite being infected with the same virus. 13 16 This points to host-specific factors, particularly the robustness of the initial mucosal innate immune response in the gut, as a decisive event in containing the virus and preventing systemic dissemination. 16 Furthermore, the emergence of new, distinct E-11 recombinant variants, such as the “Lineage 1” identified in the 2022 to 2023 French outbreak, has been linked to a dramatic increase in fatal neonatal infections, suggesting that genetic changes in the virus may confer a hypervirulent phenotype. 3 This underscores the critical need for ongoing molecular surveillance. 12 17

Management of severe neonatal E-11 remains largely supportive, requiring intensive care to manage failing organ systems. A systematic review found empiric antibiotics were used in over half of severe cases (53.6%), reflecting the difficulty in distinguishing from bacterial sepsis. Blood products are frequently essential for managing coagulopathy. IVIG was administered in about 40.9% of severe cases reviewed, often at doses of 1 to 2 g/kg ^2^ . While some evidence suggests potential benefit if given early (within 3 days of onset), 18 overall efficacy remains debated and likely depends on the specific antibody content of the IVIG preparation against the infecting strain. 1 Specific antivirals are limited; pleconaril showed potential trends towards benefit in a trial but is not widely available systemically, 19 whereas pocapavir has been used under investigational protocols with some reported success. 2 3 13 Advanced organ support including mechanical ventilation (∼21.5% of severe cases), ECMO (∼18.1%, often for myocarditis), and CVVH/dialysis may be necessary but are associated with severe disease. 2

This case also underscores the obstetric dilemma of managing a febrile pregnant woman near term. While delaying delivery might be theoretically beneficial to allow for maternal antibody production and transfer if a viral infection is recognized, clinical scenario often precludes this option. 1 As demonstrated in this case, the patient was already in active labor with a fetal malpresentation, demanding an emergency delivery despite the maternal fever. This scenario highlights the critical importance for clinicians to maintain a high index of suspicion for neonatal enterovirus infection in infants presenting with sepsis, particularly when born to mothers with a peripartum illness. 15 Close communication between obstetric and pediatric teams is essential in these situations to anticipate and prepare for potential neonatal complications.

## Conclusion

Neonatal E-11 infection presents a wide clinical spectrum, with outcomes critically determined by the timing and mode of viral transmission. The highest risk for a rapidly progressive, fulminant sepsis syndrome is associated with vertical transmission from a mother with an acute peripartum infection, a scenario that prevents the transfer of protective antibodies. This perinatal route is distinct from the often-milder postnatal infections, where passive immunity is protective, and from rare congenital infections that can result in fetal demise or fulminant disease at birth. This case of fatal E-11 sepsis in a late preterm neonate, characterized by multiorgan failure following maternal illness and known household exposure, tragically highlights the severe potential of this virus. Vigilance for enteroviral infection in septic-appearing neonates, appropriate diagnostic testing, aggressive supportive care, and stringent infection control practices are crucial. Continued surveillance and research into effective therapies are needed to improve outcomes for these vulnerable infants.
