# Supplementary material for: Fatal Neonatal Echovirus 11 Infection Following Maternal Peripartum Illness: A Case Report with Literature Review
Source: AJP Rep. 2025 Dec 30;15(4):e181–5. doi: 10.1055/a-2764-2405 (PMC12753186; doi:10.1055/a-2764-2405)
Supplement: Supplementary file 1 — Supplementary Material [file 10-1055-a-2764-2405_27799138.pdf]

Table S1 Diagnostic Criteria for Enterovirus-Associated Neonatal Hemorrhage-Hepatitis Syndrome

Diagnostic Domain	Criteria/Findings
Clinical Presentation	Acute onset in neonate; jaundice; lethargy; shock; multi-organ failure
Laboratory Findings	Markedly elevated transaminases; hyperbilirubinemia; coagulopathy (prolonged PT/INR, low fibrinogen, thrombocytopenia); disseminated intravascular coagulation
Hemorrhagic Manifestations	Diffuse hepatic and adrenal hemorrhagic necrosis; possible bleeding in other organs (e.g. renal, pulmonary, intracranial)
Pathology	Massive hepatic necrosis; adrenal hemorrhagic necrosis; intravascular coagulation, veno-occlusive changes
Virology	Enterovirus infection confirmed by PCR or viral culture from blood, liver, or other tissues
Exclusion of other causes	No evidence of other viral hepatitis (A-E), metabolic, or autoimmune liver disease

References:

1. Hirade T, Abe Y, Ito S, et al. Congenital Echovirus 11 Infection in a Neonate. Pediatr Infect Dis J 2023;42(11):1002-1006
2. Novazzi F, Piralla A, Perniciaro S, et al. A new case of Echovirus 11 neonatal fulminant hepatitis involving male twins in a Northern Italy Tertiary University Hospital: Insight on a possible immunological clue. IJID Reg 2024;12:100411
